# Comparative Analysis of Triglycerides From Different Regions and Mature Lactation Periods in Chinese Human Milk Project (CHMP) Study

**DOI:** 10.3389/fnut.2021.798821

**Published:** 2021-12-23

**Authors:** Huiquan Zhu, Aimei Liang, Xiaodan Wang, Wenyuan Zhang, Yumeng Zhang, Xiangyu He, Ying Liu, Shilong Jiang, Jing Lu, Jiaping Lv

**Affiliations:** ^1^Institute of Food Science and Technology, Chinese Academy of Agricultural Science, Beijing, China; ^2^Peking University Health Science Center (PKUHSC)-China Feihe Joint Research Institute of Nutrition and Healthy Lifespan Development, Beijing, China; ^3^Nutrition and Metabolism Research Division, Innovation Center, Heilongjiang Feihe Dairy Co., Ltd., Beijing, China; ^4^School of Food and Health, Beijing Technology and Business University, Beijing, China

**Keywords:** human milk, mature milk, lactation period, triacylglycerols, regions

## Abstract

The kinds and proportions of triglycerides of human mature milk play an independent role in the growth of infants. In this study, the human milk samples obtained from eight different Chinese cities (Chengdu, Weihai, Lanzhou, Jinhua, Beijing, Guangzhou, Zhengzhou, and Harbin) and six sequential mature lactation times (30, 60, 90, 120, 150, and 180 days) were detected for the triglycerides. The result demonstrated that total 66 triglycerides were detected in mature human milk, with acyl carbon number (ACN) numbers were locating in the range of 34–54 and double bond (DB) numbers were locating in the range of 0–6. In addition, the percentage of OPO, OPL, and OOO was relatively higher than others, accounted for more than 4% of total triglycerides in all the lactation areas and times, and the percentage of U2S and LLL triglycerides was also richest in mature milk. Furthermore, it was obvious that lactation regions had more significant effect on the triglycerides compared with lactation time and the triacylglycerols (TAGs) of human milk in Guangzhou were clearly different from that in other regions. Therefore, the results of this study will provide data reference for the design of infant formula suitable for Chinese babies.

## Highlights

- A large number of mature human milk samples from eight cities during different mature location periods were first analyzed in China.
- The kinds and proportions of triglycerides in human mature milk mainly impacted by daily eating habits of local people.
- The percentages of U2S-TAG and LLL-TAG were richest in mature human milk.

## Introduction

It is undoubted that human milk is the most beneficial nutrient food for the growth of infants and the percentage of fat in human milk accounts for about 3–5%. The triacylglycerols (TAGs) are the main components of human milk fat and the proportion of it may take up more than 98% in total fat (1). To be specific, the TAGs are the primary source of energy and provide essential fatty acids for newborns; it was reported that the TAGs accounted for ~50% of total energy of intake for newborns (2). In addition, some studies reported that the structures of the TAGs were able to affect the digestion and absorption of milk fat (3). In human milk, saturated fatty acids (S) were mainly located in sn-2 position in the TAGs and sn-1 and sn-3 positions were mainly occupied by unsaturated fatty acids (U). This USU-type TAGs can facilitate the saturated fatty acids turned into sn-2 monoglycerides to absorbing in small intestine and avoid releasing free saturated fatty acids, which could combine calcium to form unabsorbable calcium soap. Therefore, the studies focused on the structure of the TAGs that will improve our understanding of human milk and further imitate it in infant formula.

In the recent years, with the development of chromatography and mass spectrometry, the structure and composition of triglyceride in breast milk fat have attracted much attention and obtained many analyses. Morera et al. (4) reported that the proportions of some TAGs including LaMO, CaPO, LaCaO, LaPCa, LaOL, MPLn, LLO, LaOO, MPL, and MOL were stable in the mature human milk and presumed that these TAGs played the important role in physiology for infants. Another study conducted by Zou et al. (5) analyzed 45 human milk samples and the result displayed the contents of POO and POL in colostrum was significantly higher than that in transitional and mature milk in Denmark. Furthermore, total 56 TAGs were explored in human milk and infant formula and they found that the TAGs of human milk had smaller molecular weight, with 728–952 Da, compared with that of infant formula (6). A recent literature demonstrated that there were some unique TAGs constituted with C26:0 and C26:1 of mature human milk in Gambia and the difference of the TAGs in human milk produced by breasts (7).

As for Chinese human milk, some preliminary works on the TAGs were carried out, with focusing the variation of the TAGs in colostrum, transitional and mature milk. Some researchers reported that it was obvious that the proportion of the high-molecular-weight TAGs in mature milk was higher than that in the colostrum and transitional milk. On the contrary, the share of the low-/mediate-molecular-weight TAGs was higher than that in mature milk (8, 9). In addition, the differences of the TAGs of human milk collected from different cities were also found by several groups (9–11). It is common knowledge that the infants were recommended to provide human milk in the first 6 months of lactation; therefore, the composition of human milk during this period will provide more comprehensive information for designing of infant formula. However, to the best of our knowledge, the changes of the TAGs were not studied carefully during the first 6 months of lactation and different lactation regions.

In order to investigate the effect of regions and different lactation times on the TAGs composition of human milk, total 201 milk samples were obtained from 1st to the 6th month of lactation and different regions in this study including Northeast China (Harbin), East China (Weihai, Jinhua), North China (Beijing), Central China (Zhengzhou), South China (Guangzhou), Northwest (Lanzhou), and Southwest (Chengdu). Hopefully, these study data will help better understanding to the nutritional value of human milk and better design infant formula for targeted infant population.

## Materials and Methods

### Chemicals

Internal standard, 1,3(d5)-diheptadecanoyl-2-heptadecenoyl-glycerol [d5-(17:0/17:1/17:0) TAG], was purchased from Avanti Polar Lipids (Birmingham, Alabama, USA) and methanol (MeOH), acetonitrile, isopropanol (IPA), chloroform (CHCl_3_), formic acid, and ammonium formate were bought from Fisher Scientific (Pittsburgh, Pennsylvania, USA).

### Human Milk Samples

The Chinese Human Milk Project (CHMP) study, containing 1,800 volunteers who came from eight different cities located in mainland China (including Guangzhou, Weihai, Chengdu, Beijing, Harbin, Jinhua, Zhengzhou, and Lanzhou), was to evaluate the human milk composition in Chinese population (NCT03675204).

In this study, the human milk samples were obtained from different mature lactation times (30, 60, 90, 120, 150, and 180 days) after delivery (12). To be specific, the individual human milk was acquired at 9:00–11:00 a.m. in the morning before breastfeeding and the sampling time. Moreover, the samples were collected from both the left and right sides of the fully breast and the least volume of every sample was 60 ml. As for the physical condition of volunteer mothers (25–35 years old), they all were the long-term residents, had typical eating habits and healthy body, and no smoking and drinking habit. Mothers were required to wash their hands carefully with soap and water before expressing milk from breast with a human milk pump into a sterile hard plastic container; all of them had received detailed information about this study and provided a written informed consent before the beginning of this study. After collecting of human milk, samples were kept freezing at −20°C until delivered to the laboratory and then distributed and stored at −80°C for subsequent analyses.

### Extraction of Human Milk Lipids

The Folch method was used to extract the lipid from human milk (13). In short, added 3 ml of human milk to 10 ml of extracting solution, which was mixed by chloroform and methanol (2:1, v/v). In the next step, the mixture was shaken by vortex mixer for 1 min and centrifuged subsequently at 3,000 rpm for 5 min. After that, the chloroform layer was collected and re-extract the liquid supernatant with 10 ml of extracting solution in two times. Finally, the organic phase was evaporated using nitrogen blowing and stored at −80°C for further analysis.

### Triacylglycerol Composition Detected by LC-MS/MS

As for the detection method of triglycerides, the milk lipid was dissolved by the mixed liquor composed of CHCl_3_ and MeOH (1:2, v/v) to the 5 mg/ml. At the second stage, the sample solution was diluted 50 times by MeOH, which contained 10 mmol ammonium formate and 0.1% formic acid, then 20 μl diluted solution and internal standard were pipetted into the LC injection vial. The setting conditions of liquid chromatography were as follows: the mobile phase A was blended by acetonitrile and water (1:1, v/v) and mobile phase B was mixed by IPA and acetonitrile (9:1, v/v); all of them contained 10 mmol ammonium formate and 0.1% formic acid. The lipid was transferred by a BEH-C18 reversed-phase column (1.7 μm, 2.1 mm inner diameter × 100 mm). In the whole process, the column maintained at 60°C and the flow velocity was 0.3 ml/min. The sample solution was detected in the positive ionization mode and the sprayer and the solvent removal gas were nitrogen.

Specific elution conditions and mass spectrometry parameters were set according to the previous literature by Zhao et al. (8). To be specific, the whole process began with the scan pattern used to acquire the parent ion, with the 100 V in the declustering potential, the range from 400 to 1,200 m/z in mass, and 1,000 Da/s in scanning rate. In the next stage, the specific instrumental method included multiple reaction monitoring (MRM) (Q1 = Q3), information-dependent acquisition (IDA), and enhanced product ion (EPI) that was setup and applied to identify the particular fatty acids. Finally, the MRM (Q1 ≠ Q3) was chosen to quantify the TAGs constituted with special fatty acids. As for the structural isomers of the TAGs, they were distinguished by the M/Z (mass/charge) ratio and abundance.

### Statistical Analysis

The SPSS 24.0 (version 24.0, SPSS, Chicago, Illinois, USA) was used to analyze the difference of the TAG among different human milk samples, which was expressed by mean and SD. The normality and homogeneity of variance were assessed by the Shapiro–Wilk and Levene's test, respectively. The Bonferroni and Dunnet's *T post-hoc* tests were adopted to identify the difference when variance did or did not satisfy the condition of homogeneity. The partial least squares discriminant analysis (PLS-DA) was selected to further analyze the difference of human milk samples by using SIMCA-P software (version 14.1, Demo Umetrics, Umea, Sweden, UK). In order to explain the significant changes of human milk triglyceride in different lactation times and regions, Cluster 3.0 and Java TreeView were selected to stratify and cluster the data.

## Results

### Composition for the TAGs of Mature Human Milk

As shown in Supplementary Table S1, the 66 TAGs were detected and these TAGs were classified into three groups according to the proportion. The TAGs in first group contained OPO, OPL, and OOO, with the average percentage all over 4% in different lactation regions and mature times. Among them, the maximum values of OPO, OPL, and OOO were taken up 15.7, 11.88, and 10.52%, respectively. In addition, the human milk, with maximum percentage of OPO, OPL, and OOO, displayed in Guangzhou, Beijing, and Chengdu, respectively. The second TAGs group contained OLO, LOL, LPL, OSO, SPO, OMO, POP, OLaO, PLP, OPLa, and OPM, with the content of them exceeded than 1% in total TAGs; meanwhile, the maximum content of OLO, LOL, LPL, OSO, and SPO represents more than 5%. As for the lactation regions, there were some differences among the TAGs in second group and it was clearly showed that the largest percentage of SPO, POP, OPLa, and OPM and the lowest percentage of OLO, LOL, and LPL were demonstrated in Guangzhou, while these TAGs in Jinhua and Zhengzhou show the most balanced content. Finally, the third group constituted with some TAGs that the percentage of them account for more than 0.01% in total TAGs and the number of them were 50. The relative content of some TAGs, which such as OLaL, LaOLa, OMLa, PCaLa, OPoLa, LPM, LPCa, and SOS, did not change during different lactation time and regions in third group.

Due to the difference between mature breast milk, the TAGs in this study were compared to that identified in other studies (Table 1). It was undoubted that there were six triglycerides, including OOO, OPL, OPO, OSO, POP, and SPO, recognized commonly in all the countries. At the same time, some special TAGs were observed in different countries. The PoOL, PoOO, and PoLS, which all had hexadecenoic acid in their structure, were the unique TAGs of human milk in Spain and Finland. Furthermore, it was obviously showed that the CaCaCa, MMM, and MOB were explored particularly in United States. In terms of the human milk in China, there were some specific TAGs included CaOCa, CaPL, LaCaLa, LaOLa, LLaCa, LLaLa, LML, LMLa, LPCa, LPoL, LPPo, OMPo, and OPCa, most of them contained linoleic acid (C18:2).

**Table 1:** Comparison of the content of triglycerides in mature human milk between China and other countries (%)^a^.

**TAGs**	**Country**					
	**China (this study)**	**Spain (4, 14, 15)^**b**^**	**Denmark (5)**	**America (16)**	**China (previous studies) (9–11)**	**Finland (17)**
AOP	0.096–0.247	-	-	-	-	-
CaLaPo	0.006–0.271	-	-	-	-	-
CaOCa	0.014–0.146	-	-	-	0.05–0.55	-
CaPL	0.276–0.774	-	-	-	0.91–2.5	-
LaCaLa	0.015–0.93	-	-	-	0.05–0.62	-
LaCaM	0.017–0.884	-	0.17	-	0.01–0.93	-
LaLaLa	0.051–3.085	-	0.45	0.13	-	-
LaMLa	0.092–4.394	0.64	2.93	-	0.19–2.21	-
LaOLa	0.303–1.835	-	-	-	1.10–2.65	-
LaPCa	0.033–1.199	0.26	-	-	0.6–1.17	-
LaPLa	0.132–4.826	-	-	-	-	-
LaSCa	0.037–1.074	-	-	-	-	-
LLaCa	0.107–0.406	-	-	-	0.01–1.15	-
LLaL	0.87–2.021	0.14–0.146	-	-	0.01–2.23	-
LLaLa	0.255–0.853	-	-	-	0.94–1.91	-
LLL	3.894–10.407	0.73	-	0.06	1.27–2.96	-
LMCa	0.075–0.264	-	-	-	-	-
LML	1.167–2.415	-	-	-	1.04–1.46	0.25
LMLa	0.199–0.683	-	-	-	0.74–1.5	0.47
LMO	1.709–3.076	1.13–2.77	-	-	2.74–6.06	1.6
LOL	7.092–10.707	1.65–2.22	-	1.2	4–7.29	1.23
LPCa	0.249–0.659	-	-	-	0.91–1.27	0.39
LPL	8.21–11.869	0.96–2.4	-	-	4.08–8.22	0.76
LPLn	0.741–3.057	-	-	-	-	-
LPM	1.005–2.116	1.278–1.29	1.6	-	-	-
LPoL	0.799–2.63	-	-	-	0.79–0.9	0.16
LPPo	0.582–1.579	-	-	-	1.16–1.39	0.22
MLaP	0.098–2.992	0.36	2.28	-	0.42–1.47	-
MSM	0.128–1.556	-	2.2	-	-	0.09
OCaO	0.485–2.152	0.82–0.86	-	-	0–0.51	-
OEP	0.167–1.572	-	-	-	-	-
OLaL	1.25–2.337	0.94–2.09	2.38	-	1.15–4.6	1.18
OLaO	1.918–5.595	0.92–4.74	5.96	-	1.15–2.55	2.01
OLaPo	0.13–0.514	-	-	-	-	-
OLO	8.637–13.559	2.75–5.71	-	2.41	2.74–5.66	2.95
OMLa	0.3–1.637	0.57	7.27	-	0.67–1.93	0.17
OMO	2.769–6.466	3.23	-	-	1.23–2.58	2.61
OMoP	0.073–0.716	-	-	-	-	-
OMPo	0.154–0.577	-	-	-	2.42–3.20	0.38
OOO	7.625–15.397	3.65–4.46	1.4	1.97	1.74–3.42	3.12
OPCa	0.363–1.462	-	-	-	0.78–4.59	1.4
OPeO	0.112–0.779	-	-	-	-	-
OPeP	0.033–0.226	-	-	-	-	-
OPL	11.81–13.558	9.88–18.84	16.75	10.14	5.84–24.98	5.12
OPLa	1.457–5.548	2.33	10.89	-	2.08–7.23	2.35
OPM	1.329–5.253	2.97–3.12	-	3.81	1.12–3.46	2.52
OPMa	0.1–0.695	-	-	-	-	-
OPO	14.146–22.092	20.21–27.24	20.57	9.87	3.18–13.91	10.89
OPoLa	0.934–2.15	-	-	-	-	-
OPPo	0.884–2.86	3.82	-	-	0.71–0.93	1.04
OSL	2.039–3.165	0.97–2.03	-	-	3.2–4.58	0.95
OSO	2.312–6.736	0.76–1.22	0.46	2.66	0.40–0.54	1.56
PCaLa	0.139–1.152	-	-	-	-	-
PCaM	0.091–3.554	-	-	-	-	-
PCaS	0.29–5.198	2.26	-	-	0.18–1.50	-
PLaS	0.256–3.521	1.22–1.77	-	-	0.34–1.15	0.65
PLP	2.893–4.662	-	6.81	4.4	1.93–3.86	0.74
PMP	0.296–3.743	-	1.2	-	0.54–0.72	0.54
POP	3.339–10.184	4.54–5.13	6.12	11	2.35–5.98	3.02
PPP	0.403–4.684	0.32	0.38	1.58	1.21–1.81	-
PSP	0.554–4.827	0.21–0.35	0.13	1.11	0.30–0.74	-
SMLa	0.166–3.269	-	-	-	-	-
SMP	0.191–2.131	0.39	-	-	0.17–0.8	-
SOS	0.312–1.459	0.06	-	-	0.1	-
SPO	3.421–8.96	3.47–5.06	2.29	6.66	0.97–3.73	3.71
SPS	0.37–3.264	0.06–0.157	-	0.86	0.02	-
SLL	-	2.3	-	-	2.21–3.9	0.22
SLP	-	1.12–2.51	-	9.87	2.25–3.79	0.63
CaLaO	-	0.612–0.649	0.64	-	0.46–1.84	-
CaOL	-	0.241–0.242	-	-	0.62–1.86	-
PoOL	-	0.17–0.18	-	-	-	0.72
PoOO	-	0.9–1.7	-	-	-	1.31
LaPL	-	1.61	-	-	1.55–4.06	-
LaPP	-	0.79	-	-	0.91–1.34	-
PoLS	-	5.04	-	-	-	0.08
MMLa	-	-	2.38	-	1.04–1.87	-
CaCaCa	-	-	-	0.02	-	-
MOB	-	-	-	0.01	0–0.33	-
MMM	-	-	-	1.18	-	-
MOB'	-	-	-	0.56	-	-

a *B, butanoic acid; Ca, capric acid; La, lauric acid; M, myristic acid; Pe, pentadecanoic acid; P, palmitic acid; Po, palmitoleic acid; S, stearic acid; O, oleic acid; L, linoleic acid; Ln, linolenic acid; A, arachidic acid; E, Eicosenoic acid; B′, Behenic acid; –, unknown*.

b *References for each country: Spain: Pons et al. (15); Morera et al. (4); Isabel Ten-Doménech et al. (14); Denmark: Zou et al. (5); America: Kim et al. (16); China (previous studies): Tu et al. (10); Yuan et al. (9); Chen et al. (11). Finland: Fabritius et al. (17)*.

### Impact of Different Lactation Regions and Times on the TAGs

**Figure 1:**
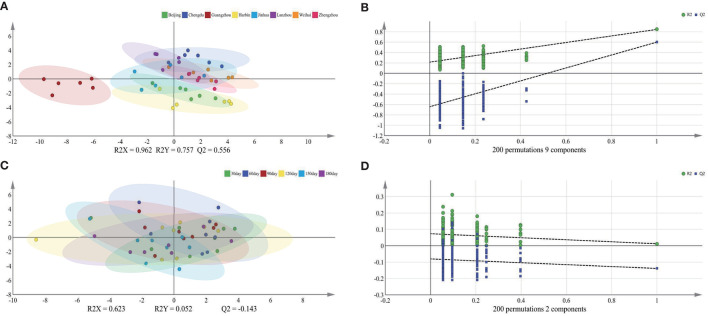
Score plots of PLS-DA **(A,C)** and goodness of the models **(B,D)** based on triglycerides of mature human milk in different lactation region and time. R2X and R2Y are the cumulative modeled variation in the X and Y matrix, respectively, and Q2 is the cumulative predicted variation in the Y matrix. The values of these parameters close to 1.0 indicate a robust mathematical model with a reliable predictive accuracy. PLS-DA, partial least-squares-discriminate analysis.

**Figure 2:**
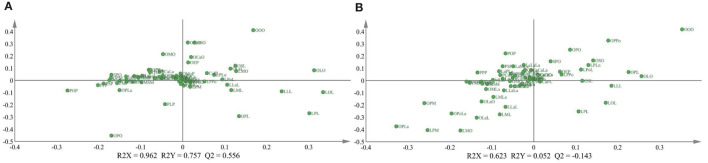
Loading plots of PLS-DA **(A,B)** based on triglycerides of mature human milk in different lactation regions and time. R2X and R2Y are the cumulative modeled variation in the X and Y matrix, respectively. Abbreviations in the triacylglycerols (TAGs) are as follows: Ca, capric acid; La, lauric acid; M, myristic acid; Pe, pentadecanoic acid; P, palmitic acid; Po, Pamitoleic acid; S, stearic acid; O, oleic acid; L, linoleic acid; Ln, linolenic acid; A, arachidic acid; E, Eicosenoic acid.

To further explore the influence of the lactation regions and times on the TAGs, the PLS-DA was selected to analyze the difference among different groups (Figure 1). The score plot C showed apparently that the lactation times, which were sequential lactation months, had no significant difference on the TAGs; moreover, this phenomenon was confirmed further by the goodness of the model (plot D). Comparing with the lactation times, the TAGs were significantly affected by the different regions, which displayed in the score plot A of Figure 1. To be specific, the TAGs in Guangzhou were obviously different with that in other cities. In other cities, the TAGs in Chengdu and Beijing also had significant differences with other regions. In addition, the loading pictures, which based on the human milk TAGs in lactation regions and time, are shown in Figure 2. Combining with the score picture (Figure 1), it was clear that some TAGs, such as PPP, PSP, OPLa and POP, had positive correlation with Guangzhou and OLO whose proportion demonstrated minimum in Guangzhou had significantly negative correlation with Guangzhou. Among other TAGs, such as OLaO, OSO, OLaL, and OSL, they had positive correlation with Chengdu and PLP negatively correlated with Chengdu. For other cities, Beijing and Harbin positively connected with OPL whose content was higher than that in other lactation regions. Finally, the PCaLa and LaPCa had negative correlation with Beijing.

**Figure 3:**
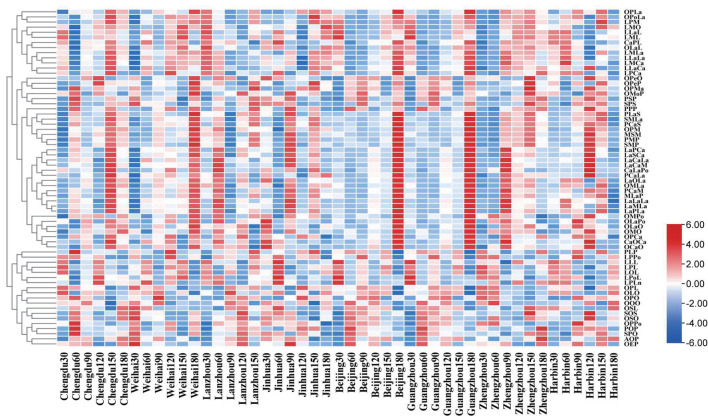
Dendrogram of the triglycerides composition in the mature human milk with different lactation regions and time clustered using the TBtools which conducted by the hierarchical clustering algorithm.

The clustering analysis shown in Figure 3 was conducted to analyze the TAGs in mature milk. The result showed that the relative share of some TAGs contained PLaS, SMLa, PCaS, OPM, MSM, PMP, SMP, PCaLa, PCaM, MLaP, OMPo, OMO, OPCa, and so on increased apparently and reached a peak at the lactation times (90–180 days) in almost all the regions. The fatty acids with the most contents in these triglycerides were oleic acid (C18:1) and myristic acid (C14:0); moreover, the TAGs containing C18:1 and C14:0 can account for more than 65%, respectively, among them.

### Characteristics of the TAGs During Different Lactation Times and Regions

**Figure 4:**
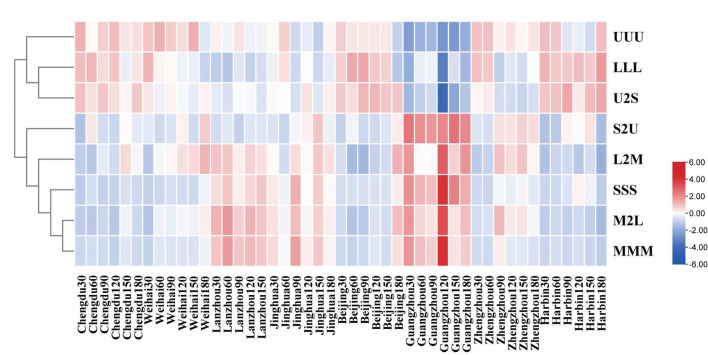
Dendrogram of characteristics of triglycerides in the mature human milk with different lactation region and time clustered using the TBtools which conducted by the hierarchical clustering algorithm.

The kinds of the TAGs in human milk were mainly distinguished by the length of chain and saturation of fatty acids. In terms of the saturation of the TAGs, the average percentages of U2S in the TAGs were highest in the mature human milk, followed by UUU, S2U, and SSS TAGs (Supplementary Table S2). In addition, the largest percentage of the TAGs divided by the length of the fatty acid was LLL, followed by L2M, M2L, and MMM, respectively (Supplementary Table S3). Then, the clustering analysis was carried out to observe the dynamic changes of characteristic in the TAGs during different lactation regions and times (Figure 4). It was noticeable that the percentages of S2U, L2M, SSS, M2L, and MMM in Guangzhou and Lanzhou were higher than other regions; furthermore, the maximums of these TAGs all occurred in the 120 day of lactation time in Guangzhou. The percentages of UUU, LLL, and U2S in all the lactation regions were similar, except that in Guangzhou which has lowest percentage.

**Figure 5:**
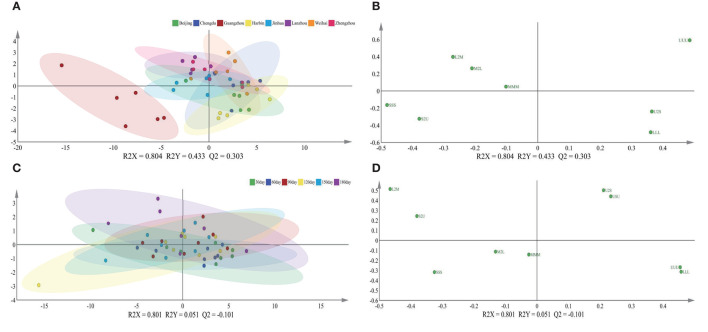
Score plots **(A,C)** and loading plots **(B,D)** of PLS-DA based on triglycerides of mature human milk in different lactation regions and time. R2X and R2Y are the cumulative modeled variation in the X and Y matrix, respectively, and Q2 is the cumulative predicted variation in the Y matrix. Abbreviations in the triglycerides are as follows: S, saturated fatty acid; U, unsaturated fatty acid; M, medium-chain fatty acid; L, long-chain fatty acid; S, short-chain fatty acid.

The relationship between the feature of the TAGs and influencing factors, which contained lactation geographic position and times, was ambiguous, so the PLS-DA approach was applied to make the further analysis. The score graph A which regarded the lactation cities as the independent variable displayed that there had significant gap in the sample between Guangzhou and other cities in Figure 5; this phenomenon revealed that the significances in lactation regions were obvious. Moreover, combing with the loading graph B, the SSS and S2U had positive correlation with Guangzhou, the U2S and LLL had positive correlation with Harbin and Beijing, respectively. As for the different mature lactation times, it displayed noticeably that there were no apparent differences among them in the graphs C and D, which were obtained by treating lactation times as the independent variable.

## Discussion

There were few studies investigated the composition and variation of the TAGs from human milk in different mature lactation times and regions in China. In this study, a total of 66 TAGs, with the ACN in the range of 34–54 and DBs number 0–6, were detected and the proportions of OPO, OPL, and OOO were highest among these TAGs. This result was same to previous study by Zhao et al. (8); however, it was slightly different from study by Yuan et al. (9) who found that the percentage of OPO, OPL, and OLL was largest in mature human milk. Meanwhile, the consequence of this study showed obviously that the percentage of OOO overtaken that of LOL during the mature lactation times in all the regions, except the lactation times included 30 and 60 days in Harbin. This phenomenon was similar with the results of the previous study by Zhao et al. (8), although Yuan et al. (9) and Zhang et al. (18) analyzed the human milk from Beijing and Finland, respectively, and they found the percentage of OOO was less than LOL; this phenomenon among these studies was likely due to the differences among individual samples and lactation regions (17).

As for the structure of the TAGs in the human milk, due to the demand of accurate nutrition, more and more researchers pay attention to the position of palmitic acid (C16:0) in the TAGs. There were some literatures demonstrated that the percentage of C16:0 was dominantly situated in the sn-2 position, with the range of 71–76% (17, 19) and C16:0 located in the side chains was easily hydrolyzed in the intestines and formed insoluble calcium soaps, which might cause constipation in body (3). Therefore, there were some studies concentrated on the special construction of the TAGs, which was unsaturated fatty acid (UPU)—palmitic acid. It was undoubted that the proportion of OPO and OPL was the highest UPU type TAGs in human milk. In this study, the percentage of OPL with the range of 11.81–13.558% located in the scope (5.84–24.98%) detected formerly in Chinese human milk; by contrast, the percentage of OPO was slightly higher than that in previous studies and the difference between them might cause by the daily diets of mother and different detecting methods. As for other UPU type TAGs, the LPLn had not been reported in previous studies (9, 11, 18), with the percentage in the range of 0.74–3.06%. Furthermore, in the residual UPU type TAGs, LPL had a relatively higher percentage (8.21–11.869%), followed by OPPo (0.884–2.86%) and LPPo (0.582–1.579%); this result was similar with preceding literature conducted in Chinese human milk (10).

The fatty acids of human milk lipid were easily affected by the age, parity, blood type, and daily diets of mother. In terms of the degree of fatty acid saturation, the fatty acids in breast milk were significantly affected by regional diets (20, 21). In this study, the proportion of U2S TAGs accounted for more than 35% in all the cities and lactation months, followed by that S2U, UUU, and SSS, respectively; this result was similar with previous studies, which conducted in Beijing and Wuxi, respectively (8, 9). The eight cities studied in this study could be divided into three categories according to the daily diet. To be specific, the daily diet of local people in Weihai, Jinhua, and Guangzhou (22) mainly focused on coastal and fresh water products. However, some cities, such as Chengdu, Zhengzhou, and Lanzhou, are located in the mid-west of China and local residents preferred to choose noodles. As for the people who lived in the north region of China, which liked Harbin and Beijing, they given priority to the rice and animal meat as the staple food (23). It was apparent that the average percentages of the U2S TAGs in Harbin and Beijing were higher than that in other cities, with the maximum 51.028%. This phenomenon might cause by the unsaturated fatty acids in mature breast milk that were dominated by oleic acid and linoleic acid (24) and animal lipids were the main source of oleic acid. At the same time, linseed oil, which was an important source of linoleic acid, was widely used in the north China.

As for the chain length of the fatty acid, it was apparent that the long-chain fatty acids (C14:C20) were the important component of the human milk TAGs in this study, with every TAG molecule containing almost long-chain fatty acids. To be specific, we found that the percentage of LLL type of the TAGs was the highest in Harbin and Beijing than in other cities, which could reach the peak 89.39 and 88.48%, respectively; however, the content of LLL was the lowest in Guangzhou, which was 69.99% at 120 days of lactation times. The previous literatures reported that the LLL triglycerides of mature human milk in Wuxi was 65.98% and it might be due to the fact that Wuxi located in southern China and relies on rice as staple food (9). Moreover, the medium-chain fatty acids (MCFAs) were synthesized endogenously when refining carbohydrates entered the body, so it might reduce the chances that long-chain fatty acids were bound to the TAGs (25) and preceding study reported the MCFAs almost located in every TAGs molecule (19). However, the percentages of TAGs include MMM, M2L, and L2M that were relatively low in this study, with about 0.2, 3, and 11%, respectively; the differentiation between these studies might cause by the different detection method and sources of sample. Another thing worth mentioning was that due to the low percentage of the TAGs could not detect by the current technology, there was no TAGs contained the short-chain fatty acids (SCFAs) in this study, which were easily digested in human body (26). Therefore, the new technologies should be created to solve this restriction in the future.

Finally, there were some special fatty acids detected in the TAGs of human milk in different country. The PoOL, PoOO, and PoLS were specifically the unique TAGs in Spain and Finland (Table 1) and C16:1 was especially monitored in these TAGs, which might be related to the local diets including the dairy products, meats, and olive oil (14, 17). However, the special TAGs in Chinese human milk contained more linoleic acid (C18:2); this was because that some vegetable oils such as colza oil, soybean oil, and peanut oil were mainly used in Chinese daily diets (22, 27). Furthermore, a recent study had reported that the triglyceride with hexacosanoic acid (C26:0) and hexacosenoic acid (C26:1) was detected in breast milk from Gambia, which has not been reported before in human breast milk (7). It has provided sufficient evidence that triglycerides of human milk are affected by geographic and dietary diversity.

## Conclusion

In conclusion, 66 TAGs were monitored in this study and the percentages of OPO, OPL, and OOO were highest in Chinese mature human milk, with all accounting for more than 4% in mature human milk. The types of the TAGs were particularly important for the development of infant formula; in terms of chain saturation of the TAGs, the proportion of U2S TAGs was largest, which all taken up more than 35% in mature breast milk. In addition, the percentage of LLL TAGs, which classified with the length of chain, represented more than 80%. At the same time, we found that compared with the lactation times, the lactation regions had significant impact on TAGs and the kinds of the TAGs were significantly different between lactation cities; this result would provide guidance for the further development of infant formula in China.

## Data Availability Statement

The raw data supporting the conclusions of this article will be made available by the authors, without undue reservation.

## Ethics Statement

The studies involving human participants were reviewed and approved by the Ethics Committee of the National Library of Medicine, Beijing. The patients/participants provided their written informed consent to participate in this study.

## Author Contributions

SJ, JLu, and JLv contribute to the conceptualization and validation, project administration, and funding acquisition. HZ and XW contribute to the methodology. AL and YZ contribute to the software and investigation. WZ, XH, and YL contribute to the formal analysis. JLu and JLv contribute to the resources and supervision. HZ contributes to the data curation, writing—original draft preparation, and visualization. HZ, AL, SJ, JLu, and JLv contribute to the writing—review and editing. All authors have read and agreed to the published version of the manuscript.

## Funding

This study was supported by the Bai-Qian-Wan Engineering and Technology Master Project (Grant #2019ZX07B01, funded by the Government of Heilongjiang Province of the People's Republic of China) and the Feihe's Internal CHMP study grant.

## Conflict of Interest

AL, XH, YL, and SJ were employed by Heilongjiang Feihe Dairy Corporation Ltd. The remaining authors declare that the research was conducted in the absence of any commercial or financial relationships that could be construed as a potential conflict of interest.

## Publisher's Note

All claims expressed in this article are solely those of the authors and do not necessarily represent those of their affiliated organizations, or those of the publisher, the editors and the reviewers. Any product that may be evaluated in this article, or claim that may be made by its manufacturer, is not guaranteed or endorsed by the publisher.

## References

[1] Koletzko B. Human milk lipids. Ann Nutr Metab. (2016) 69:27–40. 10.1159/000452819 28103608

[2] Hamosh M Bitman J Wood DL Hamosh P Mehta N. Lipids in milk and the first steps in their digestion. Pediatrics. (1985) 75:146–50. 3880885

[3] Straarup EM Lauritzen L Faerk J Høy C-E Michaelsen KF. The stereospecific triacylglycerol structures and fatty acid profiles of human milk and infant formulas. J Pediatr Gastroenterol Nutr. (2006) 42:293–9. 10.1097/01.mpg.0000214155.51036.4f 16540799

[4] Morera S Castellote AI Jauregui O Casals I Lopez-Sabater MC. Triacylglycerol markers of mature human milk. Eur J Clin Nutr. (2003) 57:1621–6. 10.1038/sj.ejcn.1601733 14647228

[5] Zou X Huang J Jin Q Guo Z Liu Y Cheong L. Lipid composition analysis of milk fats from different mammalian species: potential for use as human milk fat substitutes. J Agric Food Chem. (2013) 61:7070–80. 10.1021/jf401452y 23800239

[6] Liu ZQ Cocks BG Rochfort S. Comparison of molecular species distribution of DHA-containing triacylglycerols in milk and different infant formulas by liquid chromatography mass-spectrometry. J Agric Food Chem. (2016) 64:2134–44. 10.1021/acs.jafc.5b05920 26902881

[7] Koulman A Furse S Baumert M Goldberg G Bluck L. Rapid profiling of triglycerides in human breast milk using liquid extraction surface analysis Fourier transform mass spectrometry reveals new very long chain fatty acids and differences within individuals. Rapid Commun Mass Spectrom. (2019) 33:1267–76. 10.1002/rcm.8465 31009547 PMC6772081

[8] Zhao P Zhang S Liu L Pang X Yang Y Lu J. Differences in the triacylglycerol and fatty acid compositions of human colostrum and mature milk. J Agric Food Chem. (2018) 66:4571–9. 10.1021/acs.jafc.8b00868 29658706

[9] Yuan T Qi C Dai X Xia Y Sun C Sun J. Triacylglycerol composition of breast milk during different lactation stages. J Agric Food Chem. (2019) 67:2272–8. 10.1021/acs.jafc.8b06554 30706708

[10] Tu A Ma Q Bai H Du Z. A comparative study of triacylglycerol composition in Chinese human milk within different lactation stages and imported infant formula by SFC coupled with Q-TOF-MS. Food Chem. (2017) 221:555–67. 10.1016/j.foodchem.2016.11.139 27979241

[11] Chen Y Zhou X Han B Yu Z Yi H Jiang S. Regioisomeric and enantiomeric analysis of primary triglycerides in human milk by silver ion and chiral HPLC atmospheric pressure chemical ionization-MS. J Dairy Sci. (2020) 103:7761–74. 10.3168/jds.2019-17353 32622592

[12] Sari RN Pan J Zhang W Li Y Zhu H Pang X. Comparative proteomics of human milk from eight cities in China during six months of lactation in the Chinese human milk project study. Front Nutr. (2021) 414:2429. 10.3389/fnut.2021.682429 34458300 PMC8387594

[13] Folch J Lees M Stanley GHS. A simple method for the isolation and purification of total lipides from animal tissues. J Biol Chem. (1957) 226, 497–509. 10.1083/jcb.1.2.173 13428781

[14] Ten-Domenech I Beltran-Iturat E Herrero-Martinez JM Sancho-Llopis JV Simo-Alfonso EF. (2015). Triacylglycerol analysis in human milk and other mammalian species: small-scale sample preparation, characterization, and statistical classification using HPLC-ELSD profiles. J Agric Food Chem. (2015) 63, 5761–5770. 10.1021/acs.jafc.5b01158 26028153

[15] Pons SM Bargalló AC Folgoso CC Sabater ML. Triacylglycerol composition in colostrum, transitional and mature human milk. Eur J Clin Nutr. (2000) 54:878–82. 10.1038/sj.ejcn.1601096 11114685

[16] Kim K-M Park T-S Shim S-M. Optimization and validation of HRLC-MS method to identify and quantify triacylglycerol molecular species in human milk. Analyt Meth. (2015) 7:4362–70. 10.1039/C5AY00591D

[17] Fabritius M Linderborg KM Tarvainen M Kalpio M Zhang YM Yang BR. Direct inlet negative ion chemical ionization tandem mass spectrometric analysis of triacylglycerol regioisomers in human milk and infant formulas. Food Chem. (2020) 328:14. 10.1016/j.foodchem.2020.126991 32512466

[18] Zhang X Qi C Zhang Y Wei W Jin Q Xu Z. Identification and quantification of triacylglycerols in human milk fat using ultra-performance convergence chromatography and quadrupole time-of-flight mass spectrometery with supercritical carbon dioxide as a mobile phase. Food Chem. (2019) 275:712–20. 10.1016/j.foodchem.2018.09.150 30724254

[19] Kallio H Nylund M Boström P Yang B. Triacylglycerol regioisomers in human milk resolved with an algorithmic novel electrospray ionization tandem mass spectrometry method. Food Chem. (2017) 233:351–60. 10.1016/j.foodchem.2017.04.122 28530584

[20] Bokor S Koletzko B Decsi T. Systematic review of fatty acid composition of human milk from mothers of preterm compared to full-term infants. Ann Nutr Metabol. (2007) 51:550–6. 10.1159/000114209 18227623

[21] Wang L Li X Hussain M Liu L Zhang Y Zhang H. Effect of lactation stages and dietary intake on the fatty acid composition of human milk (A study in northeast China). Int Dairy J. (2020) 101:104580. 10.1016/j.idairyj.2019.104580

[22] Peng Y Zhou T Wang Q Liu P Zhang T Zetterström R. Fatty acid composition of diet, cord blood and breast milk in Chinese mothers with different dietary habits. Prostaglandins, Leukotrienes Essential Fatty Acids. (2009) 81:325–30. 10.1016/j.plefa.2009.07.004 19709866

[23] Wan ZX Wang XL Xu L Geng Q Zhang Y. Lipid content and fatty acids composition of mature human milk in rural North China. Br J Nutr. (2010) 103:913–6. 10.1017/S0007114509992455 19825220

[24] Gibson RA Kneebone GM. Fatty acid composition of human colostrum and mature breast milk. Am J Clin Nutr. (1981) 34:252–7. 10.1093/ajcn/34.2.252 7211726

[25] Gao C Liu G Whitfield KC Kroeun H Green TJ Gibson RA. Comparison of human milk fatty acid composition of women from Cambodia and Australia. J. Hum Lact. (2018) 34:585–91. 10.1177/0890334418772279 29758170

[26] Gómez-Cortés P Juárez M De La Fuente MA. Milk fatty acids and potential health benefits: an updated vision. Trends Food Sci Technol. (2018) 81:1–9. 10.1016/j.tifs.2018.08.014

[27] Xiang MY Harbige LS Zetterstrom R. Long-chain polyunsaturated fatty acids in Chinese and Swedish mothers: diet, breast milk and infant growth. Acta Paediatr. (2005) 94:1543–9. 10.1080/08035250500251601 16303692

