# Supplementary material for: Comparative Analysis of Triglycerides From Different Regions and Mature Lactation Periods in Chinese Human Milk Project (CHMP) Study
Source: Front Nutr. 2021 Dec 23;8:798821. doi: 10.3389/fnut.2021.798821 (PMC8734425; doi:10.3389/fnut.2021.798821)
Supplement: Supplementary file 2 [file Table_2.DOCX]

**Table** **S2** Characteristics sorted by fatty acid saturation of TAGs in mature human milk (%)^1^

| **Characteristic** | **Region** | **lactation time(day)** | | | | | |
| --- | --- | --- | --- | --- | --- | --- | --- |
|  |  | **30** | **60** | **90** | **120** | **150** | **180** |
| S2U^2^ | Chengdu | 18.12±1.82^Ab^ | 23.42±2.3^Bb^ | 20.17±2.11^ABb^ | 19.73±1.07^ABb^ | 22.49±2.32^Bb^ | 21.21±2.35^ABb^ |
|  | Weihai | 21.12±1.45^Ab^ | 20.04±0.57^Ac^ | 20.72±5.26^Ab^ | 22.93±1.03^Ab^ | 20.19±3.5^Ac^ | 24.78±3.08^Ab^ |
|  | Lanzhou | 20.16±0.96^Ab^ | 20.97±1.26^Ad^ | 19.38±1.47^Ab^ | 20.85±2.1^Ab^ | 20.84±2.26^Ab^ | / |
|  | Jinhua | 20.75±2.63^ABb^ | 19.64±1.76^Bd^ | 21.95±1.39^ABd^ | 22.93±3.46^ABb^ | 25.11±2.9^Ab^ | 21.53±2.7^ABb^ |
|  | Beijing | 19.43±0^Cb^ | 21.91±3.2^ABc^ | 19.9±2.81^ABb^ | 19.95±2.66^Bb^ | 20.82±2.69^ABb^ | 23.17±3.55 ^Ab^ |
|  | Guangzhou | 30.2±3.21^Aa^ | 28.56±3.87^Aa^ | 28.1±3.02^Aa^ | 28.98±2.88^Aa^ | 30.57±1.36^Aa^ | 29.04±3.31^Aa^ |
|  | Zhengzhou | 20.82±0.68^Bc^ | 20.52±1.72^Ad^ | 23.7±3.24^Ab^ | 23.57±3.24^Ab^ | 24.49±3.83^Ab^ | 23.96±2.15^Ab^ |
|  | Harbin | 18.24±2.1^Cc^ | 18.85±1.1B^Cd^ | 22.84±1.19^ABb^ | 22.22±4.69^BCc^ | 23.62±1.59^Ac^ | 17.74±1.88^Cc^ |
| SSS | Chengdu | 3.03±0.28^Ad^ | 5.02±1.77^Ba^ | 3.83±0.67^ABc^ | 3.81±0.67^ABd^ | 5.36±1.04^Bc^ | 4.35±1.37^ABd^ |
|  | Weihai | 3.71±0.66^Ad^ | 4.28±0.14^Aab^ | 5.13±2.09^Abc^ | 4.8±0.25^Ad^ | 4.44±2.14^Ac^ | 6.97±2.66^Ac^ |
|  | Lanzhou | 9.84±1.37^Ab^ | 11.37±2.17^Abc^ | 8.75±1.31^Ab^ | 10.35±3.17 ^Ab^ | 11.38±4.45 ^Ab^ | / |
|  | Jinhua | 9.08±2.8^ABb^ | 6.84±2.1^Bc^ | 13.31±6.45^Aa^ | 7.67±2.16^Bc^ | 10.9±2.74^ABb^ | 7.46±2.31^Bc^ |
|  | Beijing | 4.9±0^Cd^ | 5.09±1.51^Aa^ | 5.21±1.78^Ac^ | 5.33±1.85^Acd^ | 5.39±1.51^ABc^ | 8.82±2.62^Bbc^ |
|  | Guangzhou | 15.91±5.54^Aa^ | 12.9±7.24^Ab^ | 12.07±4.48 ^Aa^ | 25.4±18.46 ^Aa^ | 17.51±4.55 ^Aa^ | 13.56±5.66 ^Aa^ |
|  | Zhengzhou | 4.34±0.48^BCcd^ | 4.09±1.01^Cab^ | 7.13±1.6^ABbc^ | 6.3±2.03^ABCc^ | 7.23±2.84^Ac^ | 5.97±1.29^ABc^ |
|  | Harbin | 4.19±0.65^BCcd^ | 4.41±0.59B^Cab^ | 5.56±0.68^ABc^ | 8.4±6.88^BCd^ | 6.7±1.86^Ac^ | 3.22±0.73^Cc^ |
| U2S | Chengdu | 49.71±1.66^Aa^ | 48.24±1.39^ABa^ | 49.51±1.64A^Ba^ | 48.38±1.44^ABa^ | 47.41±0.95^Bb^ | 49.37±1.14^ABa^ |
|  | Weihai | 47.97±0.66^Aa^ | 46.41±0.67^Aa^ | 47.51±1.6 5^Aa^ | 46.89±0.46^Aa^ | 46.13±0.56^Ab^ | 47.48±1.42^Aa^ |
|  | Lanzhou | 46.08±0.93^Aa^ | 45.55±0.93^Ab^ | 46.93±0.85^Aa^ | 46.89±1.76^Ab^ | 45.64±1.73^Ab^ | / |
|  | Jinhua | 46.92±1.72^Aa^ | 47.03±0.91^Aa^ | 45.44±2.76^Ab^ | 48.1±1.53^Aa^ | 45.85±1.9^Ab^ | 47.56±0.43^Aa^ |
|  | Beijing | 49.24±0^Aa^ | 48.44±2.14^Aa^ | 50.25±2.61^Aa^ | 50.24±1.21^Aa^ | 49.46±1.17^Aa^ | 49.42±1.06^Aa^ |
|  | Guangzhou | 42.6±4.46^Ab^ | 44.33±4.99^Ab^ | 44.98±2.83^Ab^ | 35.47±11.98^Bc^ | 41.37±3.02^Ac^ | 43.67±2.9^Ac^ |
|  | Zhengzhou | 47.39±1.12^Aa^ | 47.76±1.26^Aa^ | 45.28±1.07^Bab^ | 45.17±0.82^Bb^ | 44.7±1.2^Bb^ | 45.31±1.19^Bb^ |
|  | Harbin | 49.51±2.53^Aa^ | 49.68±1.72^Aa^ | 51.03±0.57^Aa^ | 47.87±4.86^Aa^ | 50.02±1.17^Aa^ | 50.99±0.44^Aa^ |
| UUU | Chengdu | 29.14±2.23^Aa^ | 23.32±3.61^Bb^ | 26.48±2.61^ABa^ | 28.09±0.29^ABa^ | 24.74±2.47^ABb^ | 25.06±2.9^ABb^ |
|  | Weihai | 27.2±2.02^ABa^ | 29.27±1.38^Ba^ | 26.65±6.07^ABb^ | 25.39±0.81^ABb^ | 29.25±5.09 ^Bb^ | 20.77±4.25^Bc^ |
|  | Lanzhou | 23.92±1.4^Ac^ | 22.11±2.22^Bb^ | 24.94±1.45^Bb^ | 21.91±3.68 ^Bb^ | 22.14±5.22 ^Bb^ | / |
|  | Jinhua | 23.26±4.72^ABc^ | 26.49±3.36^Ab^ | 19.3±4.73^Ab^ | 21.3±4.27 ^Ac^ | 18.13±3.2 ^Ab^ | 23.57±4.48 ^Ac^ |
|  | Beijing | 26.43±0^Aa^ | 24.56±4.25^Bb^ | 24.65±4.59 ^Bb^ | 24.49±3.65 ^Bb^ | 24.33±3.08 ^Bb^ | 18.6±5.02^Bc^ |
|  | Guangzhou | 11.29±4.13^Ad^ | 14.21±6.32^Ac^ | 14.85±4.75 ^Ad^ | 10.15±7.12 ^Ad^ | 10.56±2.48 ^Ad^ | 13.73±6.67^Ad^ |
|  | Zhengzhou | 27.45±1.76^Aa^ | 27.64±2.14^Ab^ | 23.9±4.25 ^Ab^ | 24.96±5.18 ^Ab^ | 23.57±5.65 ^Ab^ | 24.76±3 ^Ab^ |
|  | Harbin | 28.06±4.9^Aa^ | 27.05±2.68^Ab^ | 20.58±1.26^BCb^ | 21.5±6.7^ABb^ | 19.67±2.07^Cc^ | 28.05±3.05^Aa^ |

^1^The lowercase letters indicate significant difference between different lactation regions; the capital letters indicate significant difference between different lactation time.

^2^S, saturated fatty acid; U, unsaturated fatty acid.

**Table S3** Characteristics sorted by chain length of TAGs in mature human milk (%)^1^

| Characteristic | Region | lactation time(day) | | | | | |
| --- | --- | --- | --- | --- | --- | --- | --- |
|  |  | 30 | 60 | 90 | 120 | 150 | 180 |
| L2M^2^ | Chengdu | 11.12±2.34^Aa^ | 10.71±3.55^Aab^ | 12.8±3.08^Aab^ | 11.9±1.4^Aa^ | 15.06±2.42^Aa^ | 13.39±3.36^Aab^ |
|  | Weihai | 10.73±1.55^Aa^ | 13.45±0.46^Aab^ | 13.92±4.15^Aab^ | 14.81±1.53^Aa^ | 15.07±2.33^Aa^ | 16.85±2.14^Ab^ |
|  | Lanzhou | 15.99±2.36^Ab^ | 15.88±1.48^Ab^ | 14.24±2.26^Aab^ | 15.35±2.22^Aa^ | 14.83±4.27^Aa^ | / |
|  | Jinhua | 13.87±3.51^Aab^ | 12.06±2.19^Aab^ | 14.94±2.32^Ab^ | 13.55±1.46^Aa^ | 15.71±1.29^Aa^ | 14.77±2.57^Ab^ |
|  | Beijing | 12.1±0^Aa^ | 9.9±1.98^Aa^ | 9.52±2.59^Aa^ | 11.36±3.84^Aa^ | 11.97±3.23^Aa^ | 16.81±1.12^Bb^ |
|  | Guangzhou | 18.29±2.48^Ab^ | 13.83±6.63^Aab^ | 13.84±5.84^Aab^ | 21.42±4.67^Ab^ | 15.41±3.34^Aa^ | 18.4±3.77^Ab^ |
|  | Zhengzhou | 10.94±1.25^Aa^ | 11.49±1.78^ABab^ | 16.07±1.29^Cab^ | 15.02±2.26^BCa^ | 15.98±4.63^Ca^ | 14±1.42^BCab^ |
|  | Harbin | 10.36±2.04^Aa^ | 11.55±0.99^Aab^ | 11.86±1.61^Aab^ | 10.81±1.9^Aa^ | 12.05±4.49^Aa^ | 9.13±3.98^Aa^ |
| LLL | Chengdu | 86.81±3.11^Aa^ | 87.29±4.43^Ab^ | 84.86±3.79^Aab^ | 86.2±1.99^Aa^ | 81.75±3.13^Aa^ | 84.23±4.44^Abc^ |
|  | Weihai | 87.44±1.76^Aa^ | 83.39±0.71^Aab^ | 83.08±5.05^Aab^ | 81.94±2.11^Aa^ | 81.79±3.14^Aa^ | 79.45±3.19^Aab^ |
|  | Lanzhou | 78.83±3.81^Aa^ | 78.08±2.02^Aa^ | 81.21±3.82^Aab^ | 79.42±3.08^Aa^ | 80.39±6.76^Aa^ | / |
|  | Jinhua | 81.96±5.5^Aab^ | 84.62±3.4^Aab^ | 79.93±4.47^Ab^ | 83.03±2.08^Aa^ | 79.76±1.84^Aa^ | 81.57±2.73^Aab^ |
|  | Beijing | 85.41±0^Aa^ | 88.28±2.55^Ab^ | 88.48±3.28^Aa^ | 85.86±5.2^Aa^ | 85.47±4.17^Aa^ | 78.36±1.53^Bab^ |
|  | Guangzhou | 75.65±4.08^Aa^ | 81.73±9.86^Aab^ | 82.01±8.6^Aab^ | 70±10.82^Ab^ | 80.51±5.16^Aa^ | 76.69±5.69^Aa^ |
|  | Zhengzhou | 86.49±1.85^Ba^ | 85.84±2.46^Bb^ | 79.05±1.62^Aab^ | 81.25±3.04^ABa^ | 80.15±6.04^Aa^ | 82.77±2.16^ABab^ |
|  | Harbin | 87.55±2.56^Aa^ | 85.99±1.3^Ab^ | 85.97±2.07^Aab^ | 86.73±3.39^Aa^ | 85.82±5.88^Aa^ | 89.39±4.81^Ac^ |
| M2L | Chengdu | 1.95±0.72^Aa^ | 1.83±0.79^Aa^ | 2.19±0.7^Aa^ | 1.8±0.6^Aa^ | 2.98±0.66^Aa^ | 2.24±1.04^Aab^ |
|  | Weihai | 1.72±0.21^Aa^ | 2.94±0.25^Aab^ | 2.8±0.84^Aab^ | 3.05±0.55^Aa^ | 2.94±0.73^Aa^ | 3.43±0.96Abc |
|  | Lanzhou | 4.64±1.28^Ab^ | 5.29±0.58^Ac^ | 4.02±1.39^Aab^ | 4.63±0.82^Aab^ | 4.24±2.12^Aa^ | / |
|  | Jinhua | 3.71±1.78^Aab^ | 3.02±1.04^Aab^ | 4.42±1.86^Ab^ | 3.03±0.59^Aa^ | 4.01±0.52^Aa^ | 3.43±0.47^Abc^ |
|  | Beijing | 2.34±0^Aa^ | 1.72±0.56^Aa^ | 1.86±0.66^Aa^ | 2.56±1.28^Aa^ | 2.39±0.89^Aa^ | 4.39±0.51^Bc^ |
|  | Guangzhou | 5.29±1.3^Ab^ | 3.87±2.72^Abc^ | 3.66±2.34^Aab^ | 7.22±4.87^Ab^ | 3.66±1.62^Aa^ | 4.41±1.65^Ac^ |
|  | Zhengzhou | 2.41±0.56^Aa^ | 2.53±0.68^Aab^ | 4.49±0.31^Bab^ | 3.51±0.81^ABa^ | 3.65±1.34^ABa^ | 3.05±0.7^Babc^ |
|  | Harbin | 1.96±0.47^Aa^ | 2.3±0.32^Aab^ | 2.03±0.43^Aa^ | 2.29±1.35^Aa^ | 2±1.28^Aa^ | 1.41±0.83^Aa^ |
| MMM | Chengdu | 0.12±0.06^Aa^ | 0.16±0.09^Aa^ | 0.15±0.06^Aa^ | 0.1±0.05^Aa^ | 0.21±0.05^Aa^ | 0.15±0.1^Aa^ |
|  | Weihai | 0.11±0.02^Aa^ | 0.22±0.01^Aa^ | 0.2±0.06^Aa^ | 0.2±0.03^Aa^ | 0.2±0.09^Aa^ | 0.27±0.12^Aa^ |
|  | Lanzhou | 0.54±0.23^Aab^ | 0.74±0.16^Ac^ | 0.53±0.3^Aab^ | 0.6±0.07^Aab^ | 0.54±0.41^Aa^ | / |
|  | Jinhua | 0.46±0.33^Aab^ | 0.31±0.19^Aab^ | 0.72±0.56^Ab^ | 0.39±0.13^Aa^ | 0.52±0.11^Aa^ | 0.36±0.09^Ab^ |
|  | Beijing | 0.14±0^Aa^ | 0.11±0.04^Aa^ | 0.14±0.07^Aa^ | 0.22±0.14^Aa^ | 0.16±0.07^Aa^ | 0.44±0.06^Bb^ |
|  | Guangzhou | 0.77±0.3^Ab^ | 0.57±0.54^Abc^ | 0.49±0.45^Aab^ | 1.36±1.55^Ab^ | 0.42±0.27^Aa^ | 0.51±0.32^Ac^ |
|  | Zhengzhou | 0.16±0.05^Aa^ | 0.15±0.07^Aa^ | 0.39±0.05^Ba^ | 0.23±0.09^Aa^ | 0.22±0.12^Aa^ | 0.19±0.09^Aa^ |
|  | Harbin | 0.13±0.05^Aa^ | 0.17±0.04^Aa^ | 0.13±0.04^Aa^ | 0.18±0.16^Aa^ | 0.13±0.1^Aa^ | 0.07±0.05^Aa^ |

^1^The lowercase letters indicate significant difference between different lactation regions; the capital letters indicate significant difference between different lactation time.

^2^M, medium-chain fatty acid; L, long-chain fatty acid; S, short-chain fatty acid.
